# Ultra-Broadband Refractory All-Metal Metamaterial Selective Absorber for Solar Thermal Energy Conversion

**DOI:** 10.3390/nano11081872

**Published:** 2021-07-21

**Authors:** Buxiong Qi, Wenqiong Chen, Tiaoming Niu, Zhonglei Mei

**Affiliations:** School of Information Science and Engineering, Lanzhou University, Tianshui South Road, Lanzhou 730000, China; qibx20@lzu.edu.cn (B.Q.); chenwq19@lzu.edu.cn (W.C.); niutm@lzu.edu.cn (T.N.)

**Keywords:** metamaterial selective absorber, refractory all-metal, broadband absorption, solar thermal energy conversion

## Abstract

A full-spectrum near-unity solar absorber has attracted substantial attention in recent years, and exhibited broad application prospects in solar thermal energy conversion. In this paper, an all-metal titanium (Ti) pyramid structured metamaterial absorber (MMA) is proposed to achieve broadband absorption from the near-infrared to ultraviolet, exhibiting efficient solar-selective absorption. The simulation results show that the average absorption rate in the wavelength range of 200–2620 nm reached more than 98.68%, and the solar irradiation absorption efficiency in the entire solar spectrum reached 98.27%. The photothermal conversion efficiency (PTCE) reached 95.88% in the entire solar spectrum at a temperature of 700 °C. In addition, the strong and broadband absorption of the MMA are due to the strong absorption of local surface plasmon polariton (LSPP), coupled results of multiple plasmons and the strong loss of the refractory titanium material itself. Additionally, the analysis of the results show that the MMA has wide-angle incidence and polarization insensitivity, and has a great processing accuracy tolerance. This broadband MMA paves the way for selective high-temperature photothermal conversion devices for solar energy harvesting and seawater desalination applications.

## 1. Introduction

Since Landy et al. first experimentally demonstrated metamaterial absorbers (MMA) in 2008 [1], MMA has spurred the interest of many researchers due to its extensive application value, e.g., solar–thermal conversion [2,3,4], photovoltaic [5], plasmonic sensors [6,7,8], imaging [9,10], stray light elimination [11], thermal emitter [12,13,14,15], photodetectors [16], and so on. In recent years, various light-matter interaction mechanisms are implemented to achieve strong light absorption, such as surface plasmon polariton [17,18], LSPP [19], magnetic polarization [20], and Fabry–Pérot cavity [21]. However, most MMAs face a serious problem, i.e., the bandwidth is relatively narrow, even if the narrowband absorbers [22,23,24,25] can be used as sensors [6,7,8,26], detectors [27], etc. This is far from meeting actual application requirements, especially in today’s growing world energy crisis, which is one of the most severe challenges facing mankind. Solar energy has enormous potential to replace fossil fuels as safe, clean, and pollution-free, and has a tremendous storage capacity. Therefore, broadband absorption has raised the interest of researchers.

In order to achieve broadband absorption, various structures are used, such as grating structures [28,29], nanocubes [30], nanotriangle [31], nanohole [3,32] and star structure [33], and so on. Among them, the traditional sandwich structure is mostly utilized due to its simplicity and low cost. For example, Mehmet Bağmancı et al. obtained above 91.8% absorption in the whole visible region by placing a star-shaped resonator and ${\mathrm{SiO}}_{2}$ spacer on the top of a tungsten (W) film [33]. Moreover, Lei and co-workers proposed a plasmonic metamaterial absorber composed of titanium-silica (Ti-SiO_2_) cubes and an aluminum (Al)-bottom film to obtain broadband absorption from visible light to near-infrared [30].

Recently, in order to further achieve a wider absorption bandwidth, some scholars proposed to place several different small unit structures horizontally in a unit structure or to place many unit structures alternately in the vertical direction. For instance, Han et al. realized a metamaterial graphene absorber consisting of four patch resonators so that the absorption rate exceeded 90% in the frequency range of 20.5–70 THz [34]. Abdelatif and co-workers proposed a funnel-shaped anisotropic MMA with absorption of 96% in the wavelength range of 200–9000 nm, which consisted of nickel–germanium (Ni/Ge) multilayer stacks [35]. Despite the significant development of the above method in high-efficiency broadband absorption, it is still challenging to achieve ultra-broadband absorption of metamaterial absorbers with a simple structure, low processing cost, and simple experimental processing procedures for practical applications. There are few reports on the application of ultra-wideband absorbtion in practical environments.

With the in-depth research of broadband absorber by scholars, some have applied ultra-wideband absorbers in solar thermal conversion (convert light energy into thermal energy). MMAs can achieve broadband selective absorption in the entire spectrum of the sun and, thus, can achieve a high-efficiency light-to-heat conversion efficiency. For example, Wu et al. designed a nanoporous hyperbolic metamaterial structure to achieve high-efficiency solar absorption with an average absorption rate of more than 96% in the wavelength range of 250–1560 nm, and, at the same time, the light-to-heat conversion efficiency reached 90.23% at a temperature of 373.15 K [36]. Wang and co-workers theoretically proposed a broadband refractory plasmonic metamaterial without refractory metal and realized solar energy absorption efficiency of 90.8% over the solar full-spectrum [37]. After that, Zhou et al. used a two-dimensional titanium grating structure to achieve an average absorption rate of 97.85% in the wavelength range of 200–2980 nm, and the PTCE was higher than 90% in the temperature range of 100–800 °C [28]. Although significant progress has been made in solar thermal conversion, there are still many limitations in terms of practical applications. Therefore, achieving broadband and high-efficiency solar spectrum absorptivity, high-efficiency photo-thermal conversion efficiency, high thermal stability, simple structure, and low manufacturing costs, is urgently needed.

In this paper, a kind of selective ultra-wideband MMA with an all-metal titanium(Ti) pyramid structure is designed. The absorptivity is higher than 90% in the wavelength range of 200–2620 nm, covering ultraviolet, visible, and near-infrared wavelengths, and the average absorption rate over this band reaches 98.68%. This broadband absorption is due to LSPP, the coupled results of multiple plasmons, and the high loss of the material itself, resulting in broadband and strong absorption. Through study on polarization and the oblique incidence of the MMA, we found that the MMA had the performance of polarization insensitivity and wide-angle incidence. At the same time, MMA can be used for solar thermal conversion, and the solar radiation absorption efficiency in the entire solar spectrum reaches 98.27%. In addition, due to the stable properties of the material used to its high melting point, the light-to-heat conversion efficiency of the MMA is higher than 95.88% at 700 °C. The MMA has a simple and consistent structure and only one material used for processing, which greatly simplifies the processing flow and saves processing costs.

## 2. Structure and Results

To achieve broadband and high absorption of sunlight energy in the ultraviolet, visible, and near-infrared bands, we used metallic titanium(Ti) material to make the MMA. As Ti has a high imaginary part of the dielectric constant in the infrared and ultraviolet bands, the choice of Ti is feasible for achieving broadband and high absorption in our selective MMA. Moreover, in order to achieve selective broadband absorption and simple experimental processing procedures, we propose a three-dimensional (3D) pyramidal structure, which can achieve excellent anti-reflection ability due to its gradual change of geometry. The parameters of the designed pyramidal structure metamaterials absorber were achieved by optimizing the width of the pyramidal bottom and top, the height, and the period of pyramidal structure metamaterials absorber. The implementation of the all-metal pyramid structure was obtained by nano-lithography technology and electrochemical deposition. The nano-hole array was first obtained by nano-lithography technology, then the pyramid hole model was obtained by nano-etching technology, and finally the pyramid array was obtained by electrochemical deposition.

**Figure 1:**
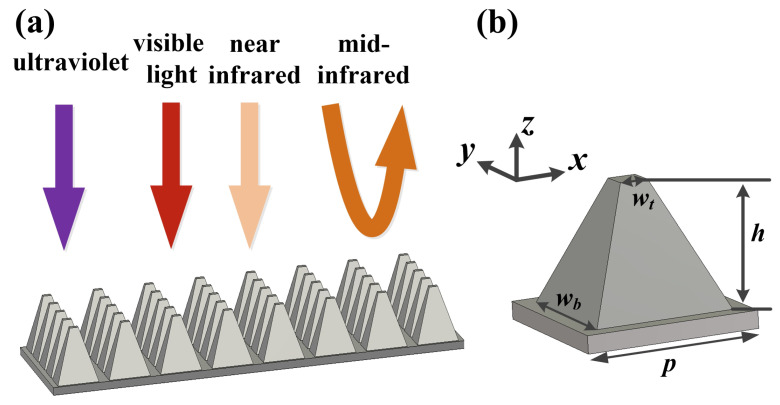
The schematic diagram of the refractory all-metal metamaterial selective absorber: (**a**) Three dimensional schematic of the MMA, (**b**) the schematic of the unit cell structure.

The 3D all-metal MMA designed is composed of periodically arranged pyramid structures, and its schematic diagram is given in Figure 1a. The unit cell of the all-metal structure MMA is shown in Figure 1b. The bottom width ($w_{b}$) is 475 nm. The top width ($w_{t}$) is set to 70 nm. The height (*h*) is 645 nm. The unit cell period (*P*) is 500 nm. The thickness of the bottom titanium film is set to 200 nm to sufficiently block light transmission.

**Figure 2:**
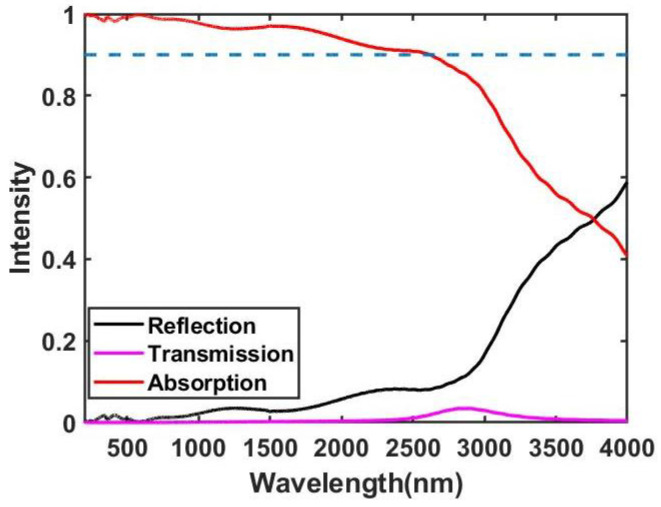
The absorption, reflection, and transmission curves of a solar energy absorber under normal incidence.

In the simulation, the 3D time-domain finite difference (FDTD) method is used to calculate the electromagnetic wave scattering parameters and electromagnetic field distribution [38]. Periodic boundary conditions in the *x* direction and *y* direction are used, and a perfect matching layer is applied in the *z* direction to remove boundary scattering. The material refractive indexes of Ti were taken from the database of Palik [39]. The absorption rate of the MMA is equal to A=1−R(Reflection)−T(Transmission). Since the metal titanium film prevents the transmission of light, the absorption rate of the absorber is A=1−R. The simulation result of the MMA is presented in Figure 2, which demonstrates an absorption rate of over 90% in the wavelength range of 200–2620 nm. The average absorption rate ($\bar{A}$) is defined to evaluate the performance of the MMA at the operating bandwidth (OBW) = $\lambda_{2}-\lambda_{1}$. The average absorption rate of the all-metal pyramid structure MMA can be expressed as:

(1) $$\bar{A}=\frac{\int_{\lambda_{1}}^{\lambda_{2}}A(\lambda )d\lambda }{\lambda_{2}-\lambda_{1}}$$

where $\lambda_{1}=200$ nm and $\lambda_{2}=2620$ nm. According to Equation (1), the average absorption rate of the MMA over this broadband reaches 98.68%, indicating near-unity perfect absorption of incident solar energy.

## 3. Principle, Analysis, and Discussions

**Figure 3:**
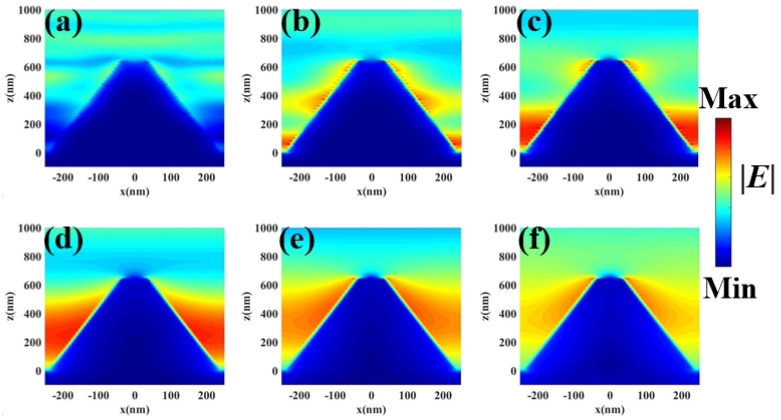
In the x–z plane, the electric field |E| distribution at wavelengths of (**a**) 254 nm, (**b**) 590 nm, (**c**) 1000 nm, (**d**)1560 nm, (**e**) 2000 nm, and (**f**) 2560 nm.

**Figure 4:**
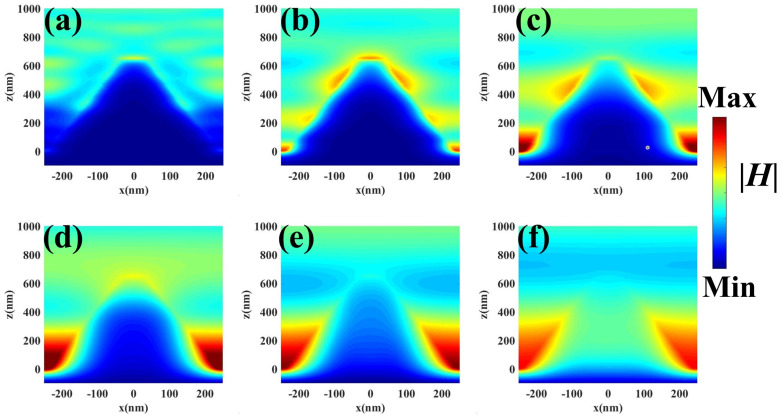
In the x–z plane, the magnetic field |H| distribution at wavelengths of (**a**) 254 nm, (**b**) 590 nm, (**c**) 1000 nm, (**d**) 1560 nm, (**e**) 2000 nm, and (**f**) 2560 nm.

To further understand the physical mechanism of the all-metal pyramid structure MMA, we give the electric field |E| and magnetic field |H| distributions under transverse electric (TE) polarization in Figure 3 and Figure 4, respectively. The position of monitor is located at y = 0. It can be seen from Figure 3a that, at the wavelength 254 nm, the local electric field is mainly located on both sides of the top of the pyramidal structure. On the other hand, as the incident wavelength changes from 590–2560 nm (Figure 3b–e), the electric field intensity gradually moves to the bottom of the pyramid structure, and the intensity of the electric field gradually increases as the wavelength increases and fills the space of the two adjacent pyramid structures. The electric field is mainly strengthened and located on both sides of the MMA pyramid structure, so that the strong absorption is caused by LSPP. In addition, the all-metal pyramid structure MMA plays an important role in the realization of the perfect absorption of the solar energy. An all-metal pyramid structure MMA is composed of resonators with different sizes from top to bottom along the *z*-axis, and thus the smooth electric field distribution between the two pyramids is caused by the mutual coupling of multiple plasmons. Moreover, Ti material itself has a large imaginary part of the dielectric constant in the infrared band; therefore, it also influences the high absorption in the infrared band. Similarly, it can be seen from Figure 4 that the magnetic field intensity also gradually increases with the wavelength, and the magnetic field intensity gradually moves to the bottom, which is consistent with the change of the electric field intensity. Therefore, the high absorption of the proposed all-metal pyramid structure MMA originates from the combination of coupled results of multiple plasmons, LSPP, and the material itself.

**Figure 5:**
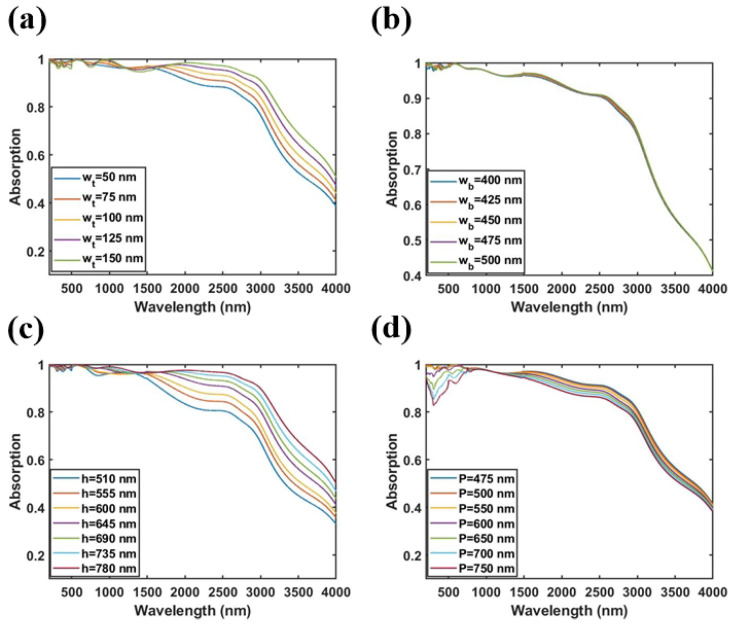
The influence of different structural parameters on the absorbing performance: (**a**) width of the top of the pyramid $w_{t}$, (**b**) width of the top of the pyramid $w_{b}$, (**c**) height of the pyramid *h*, and (**d**) period of the unit cell *P*.

Next, we study the influence of structural parameters on the absorbing performance of the MMA with the all-metal pyramid structure. In this process, the geometric parameters are as follows, $w_{t}=75\;\mathrm{nm}$, $w_{b}=475\;\mathrm{nm}$, *h* = 645 nm, and *P* = 500 nm. Only one parameter changes while the others are kept fixed for the ease of study. First, we study the influence of the top width, bottom width, and height of the all-metal pyramid structure on the absorption performance of the absorber. The simulation results are given in Figure 5a–c, respectively. It can be seen from Figure 5a–c that the absorption performance of the absorber is more sensitive to the top width and height of the pyramid structure, while the bottom width of the pyramid structure has almost no effect on the performance of the absorber. When the height of the pyramid structure is 510–780 nm, the bottom width of the pyramid structure is 50–150 nm, and the top width of the pyramid structure is 400–500 nm, the average absorption rate of the MMA in the wavelength range of 200–2620 nm exceeds 91.8%. At the same time, it can be seen that, as the height of the pyramid structure and the width of the top of the pyramid structure absorber increases, the absorption bandwidth of the MMA gradually widens, and the absorption rate in the short wavelength range does not change, while the absorption rate in the long-wavelength range gradually increases and redshifts. Thus, the coupled results of multiple plasmons can be tuned by changing the height and top width of the pyramid structure. However, it has no effect on the strong absorption caused by LSPP. The influence of the period *P* of the all-metal pyramid structured MMA on the absorbing performance of the absorber is shown in Figure 5d. It can be seen from Figure 5d that as the period *P* of the MMA gradually increases, the absorption rate in the short-wavelength range gradually decreases, and the absorption rate in the long-wavelength range gradually blueshifts, but the change is not significant. The average absorption rate in the wavelength range of 200–2620 nm is greater than 90.39%. We can also find that the period *P* of the MMA can regulate the LSPP. Generally speaking, it can be seen from Figure 5 that the average absorption rate of the MMA was greater than 90.39% within the variation range of the structural parameters as shown in Figure 5, and thus the device has great robustness in practical processing.

**Figure 6:**
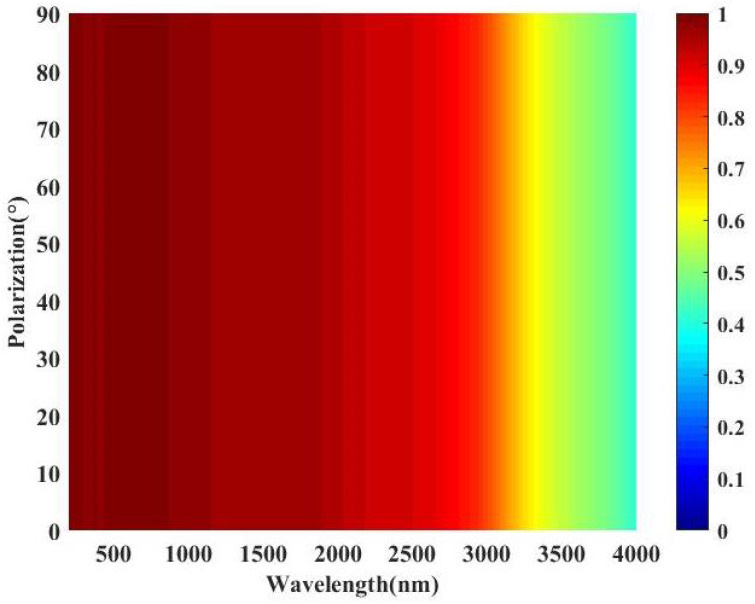
Absorption map of the proposed solar energy absorber with different polarization angles under normal incidence.

In the practical application environment, the polarization insensitivity is also a very expected performance index. We conducted a numerical simulation on the absorbing performance of the all-metal MMA with different polarization angles (0–90${}^{\circ }$), and the results are depicted in Figure 6. It can be observed from Figure 6 that the absorption performance of the MMA remains almost unchanged when the polarization angle changes in the range of 0–90${}^{\circ }$, indicating that the MMA has polarization-insensitive properties. The polarization-insensitivity performance of the MMA should be attributed to the high rotational symmetry of the MMA structure. Therefore, the all-metal MMA has polarization-insensitivity performance, which meets the polarization-insensitivity performance in practical applications.

**Figure 7:**
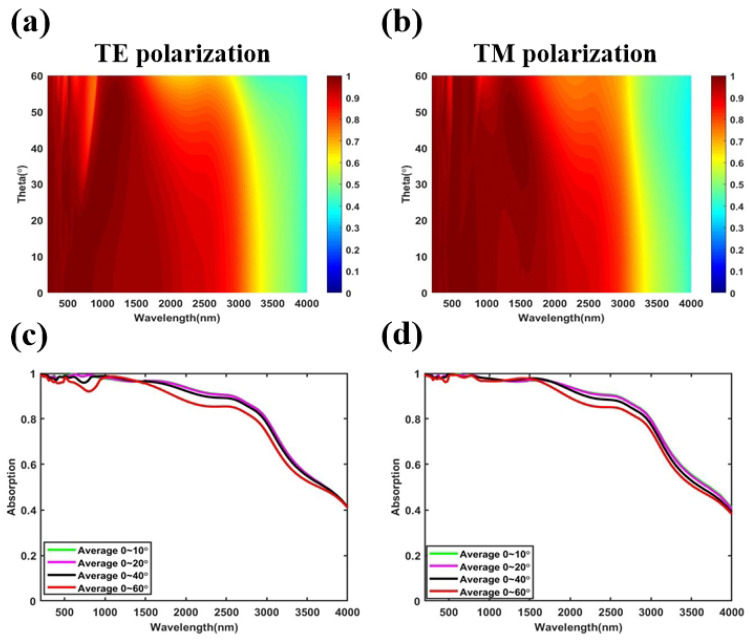
Absorption map of the proposed solar energy absorber with different incident angles under (**a**) TE polarization and (**b**) TM polarization, the average absorption for oblique incidence of (**c**) TE polarization and (**d**) TM polarization.

Oblique incidence is another important performance in the practical application environment of high-efficiency solar thermal conversion. Therefore, we study the effect of different oblique incidence angles on the absorption performance of the MMA under transverse electric (TE) and transverse magnetic (TM) polarizations, and the results are shown in Figure 7a,b, respectively. Figure 7c,d show the average absorption rates from 0–10${}^{\circ }$, 0–20${}^{\circ }$, 0–40${}^{\circ }$, and 0–60${}^{\circ }$ under the TE and TM polarizations, respectively. It can be seen from Figure 7a,b that for TE and TM polarizations, the MMA maintains a broadband and strong absorption in the range of 0–40${}^{\circ }$ in the wavelength range of 200–2620 nm. As the incident angle increases, the long-wavelength absorptivity gradually decreases. Simultaneously, it can be seen from Figure 7c,d that the TE polarization fluctuates in the short wavelength range, since the interaction between the electric field and MMA is weakened as the incident angle increases. We also calculate that, when the incident angle is 40${}^{\circ }$, the average absorption rate of TE and TM polarizations in the wavelength range of 200–2620 nm reach 93.24% and 94.29%, respectively. When the incident angle is 60${}^{\circ }$, there is a clear performance deterioration, however, the average absorption rates of TE polarization and TM polarization are 83.48% and 88.09%, respectively. Therefore, compared with other wideband absorbers with complex structures and large volumes, the all-metal pyramid structure MMA designed can also achieve good broadband wide-angle incidence absorption performance.

## 4. Solar Energy Harvesting and Conversion

In order to conduct quantitative analysis on the PTCE of the refractory all-metal MMA, we carried out a quantitative calculation of the PTCE of the absorber. According to the heat balance equation, the conversion efficiency η can be expressed as [40]:

(2) $$\eta =\frac{\alpha C*G-\varepsilon \left(\sigma T^{4}-\sigma T_{a}^{4}\right)}{C*G}$$

where α indicates the total solar absorption of the MMA, ε denotes the total thermal emissivity of the MMA, σ is the Stefan–Boltzmann quantity, *T* is the operating temperature of the MMA, $T_{a}$ is the ambient temperature, the solar radiation heat flow $G\;=\;1000\;W/m^{2}$, and *C* is the solar concentration, where *C* is generally 10–1000 [4,41,42]. In order to quantitatively determine the total solar absorption of the MMA for the solar spectrum under normal incidence, the total solar absorption (α) can be calculated by the following formula [40]:

(3) $$\alpha =\frac{\int_{0.3\mathrm{um}}^{2.5\mathrm{um}}\alpha_{\lambda }I_{\mathrm{AM}1.5}d\lambda }{\int_{0.3\mathrm{um}}^{2.5\mathrm{um}}I_{\mathrm{AM}1.5}d\lambda }$$

where $\alpha_{\lambda }$ denotes the spectral wavelength-dependent absorptivity of the MMA under normal incidence; $I_{\mathrm{AM}1.5}$ refers to the spectral intensity of solar irradiation, which is taken from global tilt AM1.5 (the actual distance of light passing through the atmosphere is 1.5-times the thickness of the atmosphere) [43,44]. The total thermal emittance of the MMA at 0.3–20 um can be calculated according to the following formula [40]:

(4) $$\varepsilon =\frac{\int_{0.3\mathrm{um}}^{20\mathrm{um}}\varepsilon_{\lambda }E_{B}\left(\lambda ,T\right)d\lambda }{\int_{0.3\mathrm{um}}^{20\mathrm{um}}E_{B}\left(\lambda ,T\right)d\lambda }$$

where $\varepsilon_{\lambda }$ represents the wavelength-dependent emittance of the MMA under normal incidence. $E_{B}\left(\lambda ,T\right)$ is the blackbody radiation spectrum at temperature *T*. According to Planck’s law, the blackbody radiation calculation formula is as follows [45]:

(5) $$E_{B}\left(\lambda ,T\right)=\frac{2\pi hc^{2}}{\lambda^{5}}\cdot \frac{1}{e^{hc/\lambda kT}-1}$$

where *h* is Planck’s constant, c is the speed of light, and *k* is Boltzmann’s constant. Based on Kirchhoff’s law, at the thermal equilibrium, the heat absorbed by an object at the same temperature is equal to the radiant heat at the same temperature.

Based on the above equation, the total solar absorption of the MMA is up to 98.27% (Equation (3)), indicating that the device has a high solar energy absorption performance. Calculating the PTCE of the absorber (Equation (2)), when the temperature is 100 °C and the solar concentration C=10, the PTCE of the MMA reaches 97.42%. In general, the photothermal conversion device works at 700 °C [46], i.e., 973.15 K, the solar concentration C=1000, and the PTCE reaches 95.87%. Based on these data, the refractory all-metal MMA has the excellent properties of a wide-angle of incidence, high efficiency of solar heat conversion efficiency, and high-temperature stability, and it has great application potential in future photothermal conversion.

## 5. Conclusions

In conclusion, we proposed a refractory all-metal pyramid structure ultra-wideband metamaterial selective absorber to achieve high-performance solar absorptions. The metamaterial selective absorber had an absorption rate of over 90% in the wavelength range of 200–2620 nm, and the average absorption rate was up to 98.68%. Through the analysis of the electric and the magnetic field distribution diagram, we concluded that the MMA achieved broadband absorption due to LSPP, the coupled results of multiple plasmons, and the strong loss of the refractory titanium material itself. Additionally, the MMA was insensitive to polarization and the angle of incidence. Since the total solar absorption rate under AM1.5 reached 98.27%, the MMA has great application value in light-to-heat conversion. At the same time, due to the high melting point of the metal titanium material, the photothermal conversion of the MMA at 700 °C was also studied, and the PTCE was up to 95.87%. Based on the large processing accuracy tolerance and simple structure of the absorber, it has great advantages in practical processing and reducing processing costs and, thus, can be processed and produced on a large scale. The MMA is only composed of metallic titanium; therefore, the processing procedure is greatly reduced. The MMA has great application potential in solar energy harvesting, seawater desalination, and solar evaporation.

## Data Availability

The data presented in this study are available on request from the corresponding author.
